# Hypertensive rats show increased renal excretion and decreased tissue concentrations of glycine betaine, a protective osmolyte with diuretic properties

**DOI:** 10.1371/journal.pone.0294926

**Published:** 2024-01-02

**Authors:** Izabella Mogilnicka, Kinga Jaworska, Mateusz Koper, Klaudia Maksymiuk, Mateusz Szudzik, Mariusz Radkiewicz, Dawid Chabowski, Marcin Ufnal

**Affiliations:** 1 Department of Experimental Physiology and Pathophysiology, Laboratory of the Centre for Preclinical Research, Medical University of Warsaw, Warsaw, Poland; 2 Mass Spectrometry Laboratory, Institute of Biochemistry and Biophysics, Polish Academy of Sciences, Warsaw, Poland; Southern University and A&M College, UNITED STATES

## Abstract

Hypertension leads to water-electrolyte disturbances and end-organ damage. Betaine is an osmolyte protecting cells against electrolyte imbalance and osmotic stress, particularly in the kidneys. This study aimed to evaluate tissue levels and hemodynamic and renal effects of betaine in normotensive and hypertensive rats. Betaine levels were assessed using high-performance liquid chromatography-mass spectrometry (HPLC-MS) in normotensive rats (Wistar-Kyoto, WKYs) and Spontaneously Hypertensive rats (SHRs), a model of genetic hypertension. Acute effects of IV betaine on blood pressure, heart rate, and minute diuresis were evaluated. Gene and protein expression of chosen kidney betaine transporters (SLC6a12 and SLC6a20) were assessed using real-time PCR and Western blot. Compared to normotensive rats, SHRs showed significantly lower concentration of betaine in blood serum, the lungs, liver, and renal medulla. These changes were associated with higher urinary excretion of betaine in SHRs (0.20 ± 0.04 vs. 0.09 ± 0.02 mg/ 24h/ 100g b.w., p = 0.036). In acute experiments, betaine increased diuresis without significantly affecting arterial blood pressure. The diuretic response was greater in SHRs than in WKYs. There were no significant differences in renal expression of betaine transporters between WKYs and SHRs. Increased renal excretion of betaine contributes to decreased concentration of the protective osmolyte in tissues of hypertensive rats. These findings pave the way for studies evaluating a causal relation between depleted betaine and hypertensive organ damage, including kidney injury.

## Introduction

Organic osmolytes such as betaine (glycine betaine, trimethylglycine, TMG), trimethylamine N-oxide (TMAO), taurine, or urea, counteract the detrimental impact of increased hydrostatic and osmotic pressure [1,2] on eukaryotic cells. Accumulation of these molecules is particularly important in organs, which are constantly exposed to hyperosmolar stress, e.g., the kidneys.

Betaine is one of the critical organic osmolytes in the kidney. In humans, betaine comes from diet or endogenous synthesis, which requires irreversible oxidation of choline, mainly in the liver and kidneys [3–7]. Primary sources of betaine in the western diet include beetroots, wheat, seafood, and spinach [3,4,8]. Betaine levels are primarily controlled by the liver metabolism and only to a small extent by urinary excretion. Specifically, it is converted to *N*, *N*- dimethylglycine and methionine by cytosolic betaine-homocysteine methyltransferase (BHMT) [9,10].

Under normal circumstances, renal clearance of betaine is low [11,12], and urinary excretion does not exceed 5% of the ingested amount [13,14], as nearly all freely filtered betaine is reabsorbed from the tubular lumen, mainly in the proximal tubule [15]. Excess urinary loss of betaine suggests pathology and has been reported in chronic kidney disease (CKD) [16] and diabetes mellitus (DM) [17–19].

Increased blood pressure and water-electrolyte disturbances in hypertension cause hydrostatic and osmotic stress to the cells. It is unclear what is the effect of these hazardous conditions on tissue distribution of protective osmolytes. Hypertension is reportedly associated with altered plasma levels of betaine in humans [20]. However, there are only a few reports on physiological levels of betaine in tissues [21,22], and none compare betaine handling in hypertensive and normotensive animals.

We hypothesized that betaine balance and its renal handling are altered in hypertensive animals. To test this hypothesis, we set three objectives for this study. The primary objective was to establish whether there is a difference in betaine concentration in tissues, blood, and urine between normotensive and hypertensive animals. Second, we aimed to examine whether betaine exerts systemic hemodynamic and diuretic effects. Finally, we compared the expression of transporters reabsorbing betaine from the renal tubule in normotensive and hypertensive animals.

## Materials and methods

### Animals

The study was performed according to the guidelines from Directive 2010/63 EU and approved by the 2nd Local Bioethical Committee in Warsaw (permission: WAW2/072/2020). Male Spontaneously Hypertensive (SHR) and Wistar Kyoto (WKY) rats (14–16 weeks old; 280–360 g b.w.) were obtained from the Central Laboratory for Experimental Animals, Medical University of Warsaw, Poland. Rats were housed in polypropylene cages (3–4 per cage), with environmental enrichment, under a daily cycle of 12 h light and 12 h darkness, temperature 22–23°C, humidity 45–55% and fed the standard laboratory diet (Labofeed B standard, Kcynia, Poland) and water *ad libitum*.

### Chemicals

Betaine anhydrous (*N*, *N*, *N*-trimethylglycine) was acquired from Sigma-Aldrich (St. Louis, MO, USA) and diluted in 0.9% normal saline for IV infusion. Urethane was purchased from Sigma-Aldrich (Poznań, Poland).

For high-performance liquid chromatography-mass spectrometry (HPLC-MS) betaine hydrochloride, *tert*-butyl bromoacetate (TBBA), ammonia solution and ammonium formate were purchased from Sigma-Aldrich (St. Louis, MO, USA). Betaine-D3 hydrochloride was obtained from Toronto Chemicals Research (North York, Canada). The stock solution of betaine was prepared fresh in methanol. LC-MS grade acetonitrile, HPLC grade acetone, HPLC grade methanol and formic acid were obtained from J.T. Baker (Phillipsburg, New Jersey, USA). Ultra-pure water (Mili-Q water) was produced by a water purification system (Mili-Q, Millipore, Milford, MA, USA).

### Betaine in body fluids and tissues

#### Experimental protocol

WKYs (n = 8) and SHRs (n = 8) were maintained for 2 days in metabolic cages to evaluate the 24-hr water and food balance and to collect urine for renal function evaluation. Samples from the second day were analyzed. Urine from voluntary micturition (spot urine) was collected directly into a plastic tube for betaine excretion studies. Animals were then anesthetized with intraperitoneal urethane (IP 1.5 g/kg b.w.), blood samples were drawn from the right ventricle of the heart, and animals were sacrificed by decapitation. Tissue samples of the liver, heart, brain, kidney medulla and cortex, colon, small intestine, and lungs were collected and stored in -80°C.

Serum creatinine was analyzed using Cobas 6000 analyzer (Roche Diagnostics, Indianapolis, USA).

#### HPLC-MS

Betaine tissue distribution was assessed using HPLC-MS. Samples for betaine distribution were prepared as follows: a 50 μl of *tert*-butyl bromoacetate in acetonitrile, 10 μl of 2.5% ammonia solution, and 50 μl of acetone (containing internal standards) were added to 20 μl of a sample (biological and calibration samples). The mixture was mixed and incubated at room temperature. After 30 min, 25 μl of 0.5% formic acid in 50% acetonitrile was added. Next, the solution was centrifuged, and an aliquot was injected into the apparatus. Tissue samples were weighed and homogenized in 10% ethanol (90 μl per 10 mg of tissue) using Precellys Cryolys Evolution tissue homogenizer (Bertin Instruments). After homogenization, samples were stored at -80⁰C until analysis.

The analysis was performed using a Waters Acquity Ultra Performance Liquid Chromatograph (Waters, Milford, Massachusetts, USA) coupled with a Waters TQ-S triple-quadrupole mass spectrometer (Waters, Manchester, UK). Waters MassLynx software (Waters, Manchester, UK) was used for the instrument control and data acquisition, and Waters TargetLynx (Waters, Manchester, UK) was used to process data. Chromatographic separation was performed using a Waters HILIC column (1.7 μm, 2.1 mm x 50 mm) (Waters, Milford, Massachusetts, USA) thermostatted at 60⁰C. Mobile phase A was 15 mM ammonium formate in Mili-Q water, and mobile phase B was acetonitrile. The flow rate of the mobile phase was set at 0.5 mL/min. The total separation time was 2.2 min. The mass spectrometer was operated in multiple-reaction monitoring (MRM)-positive electrospray ionization (ESI+) mode. Mass spectrometer optimized settings were as follows: capillary voltage = 2.5 kV, desolvation temperature = 350°C, desolvation gas flow = 550 L/h, cone gas flow = 150 L/h, nebulizer gas pressure = 7.0 bar, source temperature = 150°C. S1 Table in supplement presents MRM transitions, cone voltages, collision energies and retention times used in the described method. The first MRM transition of each compound served as a quantitative transition, the second as a confirmation transition.

Betaine concentration was estimated using a calibration curve derived from a series of calibrator samples by spiking the betaine stock solution into water. The calibration curve was generated by comparing a ratio of the betaine’s peak area to the peak of the internal standard against a known analyte concentration. Biological samples (serum, urine, tissue) were compared with an obtained calibration curve. The mean R2 coefficients of calibration curves were not lower than 0.98. The limit of quantification (LOQ) was 20 ng/ml.

#### RT-qPCR

To compare mRNA and protein levels of the two types of betaine transporters, SLC6a12 (BGT1) and SLC6a20 (SIT1), in the kidneys of SHR and WKY rats, we performed RT-qPCR and Western blot analyses.

Total cellular RNA was extracted from the kidney cortex and medulla samples with Trizol reagent (Invitrogen, Carlsbad, CA, USA) following the manufacturer’s protocol and as previously described [23]. Real-time quantitative PCR analysis was performed using Bio-Rad CFX Maestro Software (Hercules, CA, USA) with specific primers (S2 Table). The products were detected with iTaq^®^ Universal SYBR Green Supermix (Bio-Rad, Hercules, CA, USA). The products were subjected to a melting curve analysis and gel electrophoresis to confirm the specificity of amplification. Transcript levels were normalized relative to the Glyceraldehyde-3-Phosphate Dehydrogenase (*GAPDH)* reference gene.

#### Western blot

Total protein extracts were prepared from the kidney cortex and medulla to determine kidney SLC6a12 and SLC6a20 protein levels (as described previously [23]). Samples were resolved by electrophoresis on 10% SDS/PAGE gels to determine protein levels. Resolved proteins were then transferred to PVDF membranes (Bio-Rad, Hercules, CA, USA), blocked using skim milk and incubated with primary and secondary antibodies. For quantitative analysis of protein content, reactive bands were quantified relative to those of beta-actin using ChemiDoc MP Imaging System with Quantity One software (Bio-Rad, Hercules, CA, USA). S3 Table summarizes antibodies used for Western blot analyses.

### Hemodynamic and renal excretion studies

WKY (n = 25) and SHR (n = 18) rats were randomly assigned to the following experimental series:

WKY betaine 0.5 mmol/ kg, n = 6; WKY betaine 2.8 mmol/ kg, n = 6; WKY betaine 5.0 mmol/ kg, n = 7; WKY vehicle, n = 6; SHR betaine 2.8 mmol/ kg, n = 5; SHR betaine 5.0 mmol/ kg, n = 6; SHR vehicle, n = 7.

All animals were anesthetized with urethane (IP 1.5 g/kg b.w.). The femoral vein was cannulated with a polyurethane catheter for intravenous (IV) treatment and the femoral artery for BP measurement. The arterial catheter was connected to the BP and HR recording system, the Biopac MP 160 (Biopac Systems, Goleta, CA, USA). The urinary bladder was exposed with a sagittal abdominal incision and was cannulated for timed urine collection.

After the surgery, during the 30-min stabilization period, 0.9% normal saline was IV infused at a flow of 5 ml/ kg b.w./h. Next, three 10-min urine collections were taken to determine baseline excretion rates in each experimental group. Afterward, betaine (diluted in 0.9% normal saline) or vehicle (0.9% normal saline) were infused IV, first as a priming bolus dose (protocol from our previous study, betaine dosing was equimolar to TMAO [24]) for 5 min (flow rate: 5 ml/ kg b.w./ 5 min), followed by a continuous infusion for 55 min (flow rate: 5 ml/ kg b.w./ 55 min). BP and HR were recorded for 10 min at baseline (before treatment) and for 70 minutes after the start of the infusion of either betaine or vehicle. Seven 10-min urine samples were collected during the infusion of betaine or vehicle to measure minute diuresis. Analysis of solutes excretion were performed on the samples collected during the 30-min long periods. After completing the experiments, rats were euthanized by decapitation, the blood from the right ventricle was drawn, and both kidneys were collected and weighed.

The volume of the urine samples was determined gravimetrically. Excretion of total solutes (UosmV) was evaluated with the cryoscopic Osmomat 030 osmometer (Gonotec, Berlin, Germany). All variables were calculated using the standard formulas. Because kidney weights differed between WKY and SHR, measurements were then factored for kidney weights in g (UXV/g k.w.). All measurements were performed in duplicates (technical replicates).

### Statistical analyses

The sample size was calculated, assuming blood betaine concentrations in normotensive rats, as reported by other researchers [21] and in our pilot studies (data not included). We chose between-group difference in serum betaine as a primary endpoint, assuming a between-group difference of 20%, the average blood betaine concentration for the population of 195 μmol/l and common standard deviation of 40 μmol/l, the alpha error of 0.05 and test power of 0.8.

Mean arterial blood pressure (MABP) and heart rate (HR) were calculated from blood pressure tracings by Acq Knowledge 4.3.1 software (Biopac Systems, Goleta, CA, USA). The Shapiro-Wilk test was used to test the normality of the distribution. For the evaluation of changes in hemodynamic and renal parameters (UV/g k.w./min, UosmV/g k.w.) in response to betaine, baseline values were compared with values after the treatment using one-way analysis of variance (ANOVA) for repeated measures, followed by paired t-test with p-values corrected using Bonferroni’s or Tukey’s method where appropriate. Differences between the groups at the respective time-points were evaluated by ANOVA, followed by a t-test for independent variables, adjusted using Bonferroni’s correction. For differences in betaine levels, metabolic and renal function parameters, and expression of SLC6a12 and SLC6a20 transporters between WKY and SHR rats, an unpaired t-test was used. A value of two-sided p<0.05 was considered significant. Analyses were performed using RStudio Version 1.4.1106 (RStudio, PBC, Boston, MA, USA).

## Results

All raw data are available in S1 Appendix.

### Betaine in body fluids and tissues

Concentrations of betaine in systemic blood serum and tissues of normotensive and hypertensive rats are presented in Table 1. Physiological levels of betaine in the investigated tissues ranged from ~25 μM to ~2900 μM. Betaine concentration was higher in all analyzed tissues except the brain than in the systemic blood. The liver demonstrated the highest betaine concentration, followed by the renal medulla and renal cortex.

**Table 1:** Concentration of betaine in body fluids [μmol/L] and tissues [μmol/kg] (n = 6–7) in SHR (hypertensive) and WKY (normotensive) rats.

Tissue	WKY	SHR	p-value
Systemic blood serum	252.23 ± 44.01	130.92 ± 20.22	**0.041**
Urine	304.62 ± 76.53	402.45 ± 57.14	0.327
Renal medulla	2044.24 ± 157.90	1608.94 ± 87.61	**0.043**
Renal cortex	1695.68 ± 178.96	1446.77 ± 114.21	0.273
Liver	2933.08 ± 105.76	1723.43 ±182.09	**0.0004**
Brain	26.39 ± 2.70	24.05 ± 1.77	0.488
Heart	397.59 ±37.38	374.23 ± 44.04	0.695
Lungs	607.05 ± 64.20	351.23 ± 36.02	**0.009**
Small intestine	755.77± 33.72	711.61 ± 70.32	0.588
Colon	446.13 ± 77.94	467.39 ± 118.66	0.884

Betaine levels are expressed in μmol/L (systemic blood serum and urine) or μmol/kg (tissues). Presented values are means ± S.E.M, p-values by a t-test.

Normotensive rats had significantly higher betaine concentrations in renal medulla, liver, and lungs samples compared to SHR rats. The blood serum concentration of betaine in WKYs was approximately two times higher than in SHRs. 24-hour urine betaine excretion [mg/100g b.w.] was two times higher in SHR rats compared to WKY (Table 2).

**Table 2:** Metabolic and renal function parameters in SHR (hypertensive) and WKY (normotensive) rats.

Parameter	WKY	SHR	p-value
Body weight (g)	347.16 ± 12.17	352.56 ± 7.55	0.713
Food intake (g/24 h/100g b.w.)	5.99 ± 0.25	5.45 ± 0.15	0.099
Stool output (g/24 h/100g b.w.)	2.55 ± 0.15	2.80 ± 0.21	0.347
Water intake (ml/24 h/100g b.w.)	7.72 ± 0.27	10.25 ± 0.54	**0.002**
Urine output (g/24 h/100g b.w.)	2.86 ± 0.15	4.58 ± 0.21	**0.003**
Serum creatinine (mg/dl)	0.78 ± 0.10	0.93 ± 0.14	0.412
Urine betaine (mg/24 h/100g b.w.)	0.09 ± 0.02	0.20 ± 0.04	**0.036**

Presented values are means ± S.E.M, p-values by a t-test for independent variables.

#### General metabolic and renal function parameters

There was no significant difference between WKY and SHR rats in body mass, food intake, and stool output. SHR rats showed higher water intake and 24-hr urine output compared to normotensive rats. Serum creatinine was not significantly different between the groups (Table 2).

#### Renal transporters of betaine

**Fig 1:**
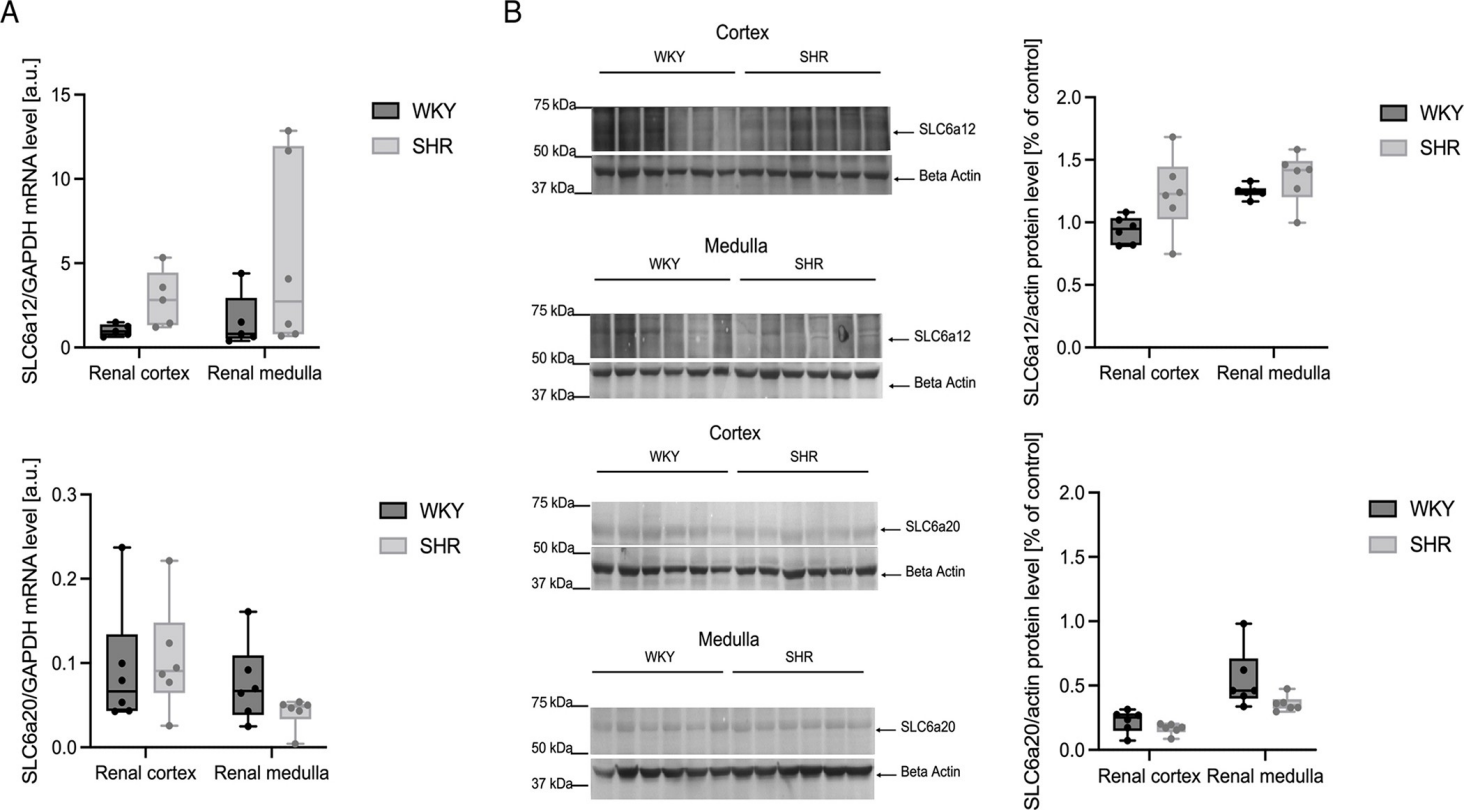
Renal betaine transporters’ genes and protein expression. (A) RT-qPCR analysis of SLC6a12 (BGT1) and SLC6a20 (SIT1) transcript levels in the renal cortex and renal medulla in SHR and WKY rats. (B) Western blot analysis of SLC6a12 (BGT1) and SLC6a20 (SIT1) protein levels from total protein extract prepared from the kidneys. A representative immunoblot is shown. Immunolabeled SLC6a12, SLC6a20, and beta-actin loading control bands were quantified using Molecular Imager. The figure presents relative levels of the test proteins and mRNA levels plotted in arbitrary units; n = 5–6 for each group.

SLC6a12 and SLC6a20 mRNAs and proteins have been detected in the kidney medulla and cortex samples of both rat strains. The RT-qPCR analysis demonstrated a tendency towards higher expression of SLC6a12 mRNA in the kidney cortex of hypertensive rats compared to normotensive animals (p = 0.069). There were no significant differences in the levels of SLC6a12 protein. There were no significant differences in the expression of SLC6a20 mRNA or protein between WKY and SHR (Fig 1).

### Hemodynamic and renal excretion studies

#### Effects of betaine on hemodynamic parameters

At baseline, anesthetized SHR rats had higher mean arterial blood pressure (MABP) and heart rate (HR) compared to WKY (MABP: 114.1 mmHg vs. 81.4 mmHg, p<0.001, HR: 312 bpm vs 276 bpm, p = 0.015). There were no significant within-group differences at baseline (S4 Table).

In WKY rats, administration of vehicle or betaine at a dose of 0.5 mmol/kg did not produce changes in MABP. Betaine at a dose of 2.8 and 5 mmol/kg caused a slight and transient increase in MABP right after the administration, followed by a decrease in MABP. However, there were no significant differences between the controls and betaine groups in other time-points.

**Fig 2:**
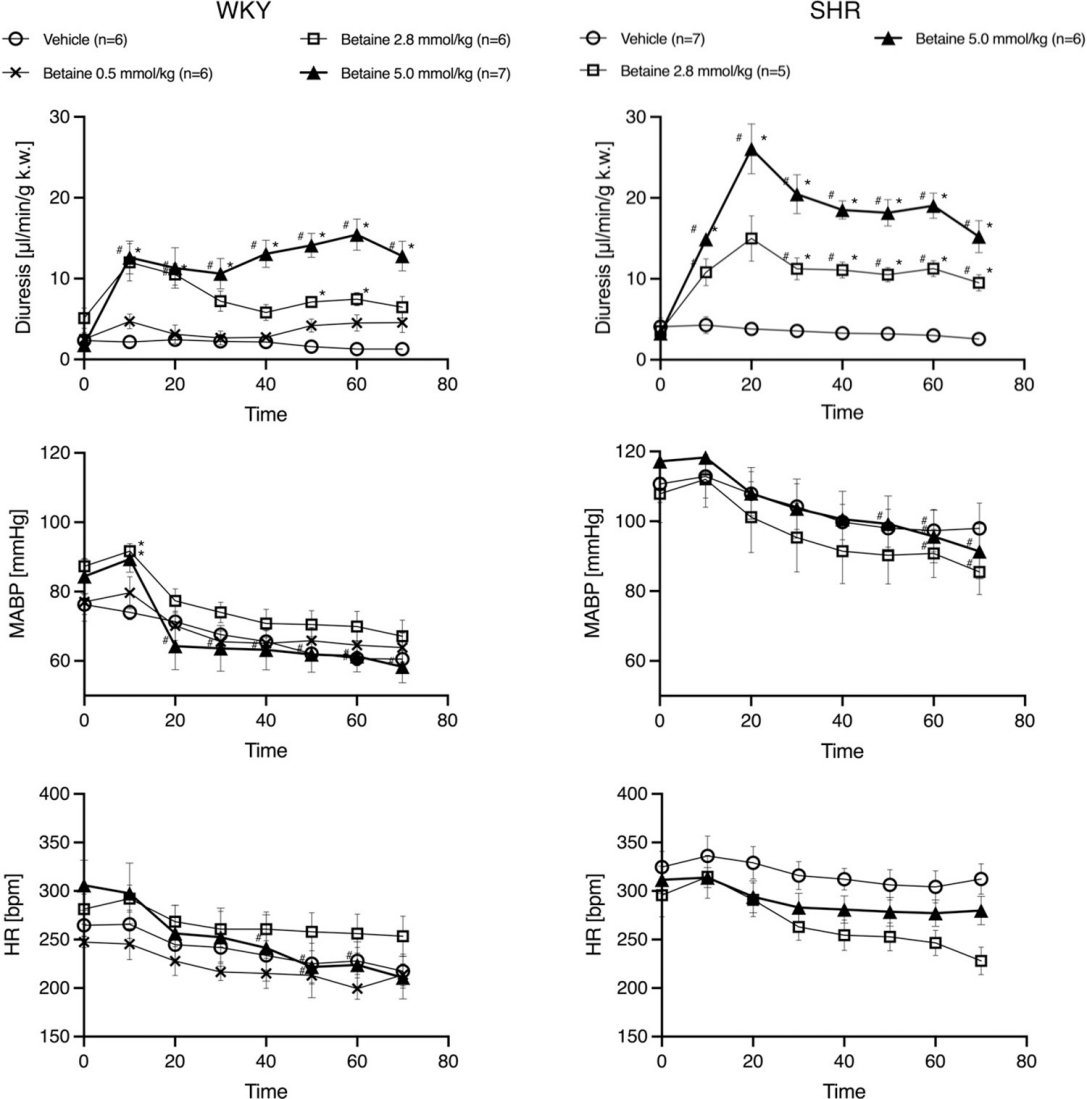
Effects of betaine on diuresis [μl/min/g k.w.], HR [bpm] and MABP [mmHg] in anesthetized Spontaneously Hypertensive rats and Wistar Kyoto normotensive rats. The priming dose of betaine was 0.5 mmol/kg b.w., 2.8 mmol/kg b.w. or 5.0 mmol/kg b.w. (5 min in a bolus), followed by continuous infusion at a rate of 0.5 mmol/kg b.w., 2.8 mmol/kg b.w. or 5.0 mmol/kg b.w./ 55min. Values are means ± S.E.M. ^#^—p<0.05 post-treatment vs. baseline, *- p<0.05 betaine vs. vehicle within the same rat strain, ANOVA for repeated measures followed by a t-test for independent variables with Bonferroni’s correction.

In SHR, administration of vehicle or betaine at any dose did not result in changes in MABP. There was no significant difference in HR after IV infusion of vehicle or betaine at any dose (Fig 2).

Detailed results are depicted in Fig 2.

#### Effects of betaine on renal excretion

Baseline minute diuresis, under anesthesia, was not significantly different in SHR and WKY rats (3.66 ± 0.54 vs. 2.89 ± 0.47 μl/min/g k.w, p = 0.285). However, at baseline, betaine excretion was higher in SHR than WKY (187.86 ± 70.86 vs. 70.17 ± 21.99 ng/min/g k.w. respectively; p = 0.002) (S1 Fig).

**Fig 3:**
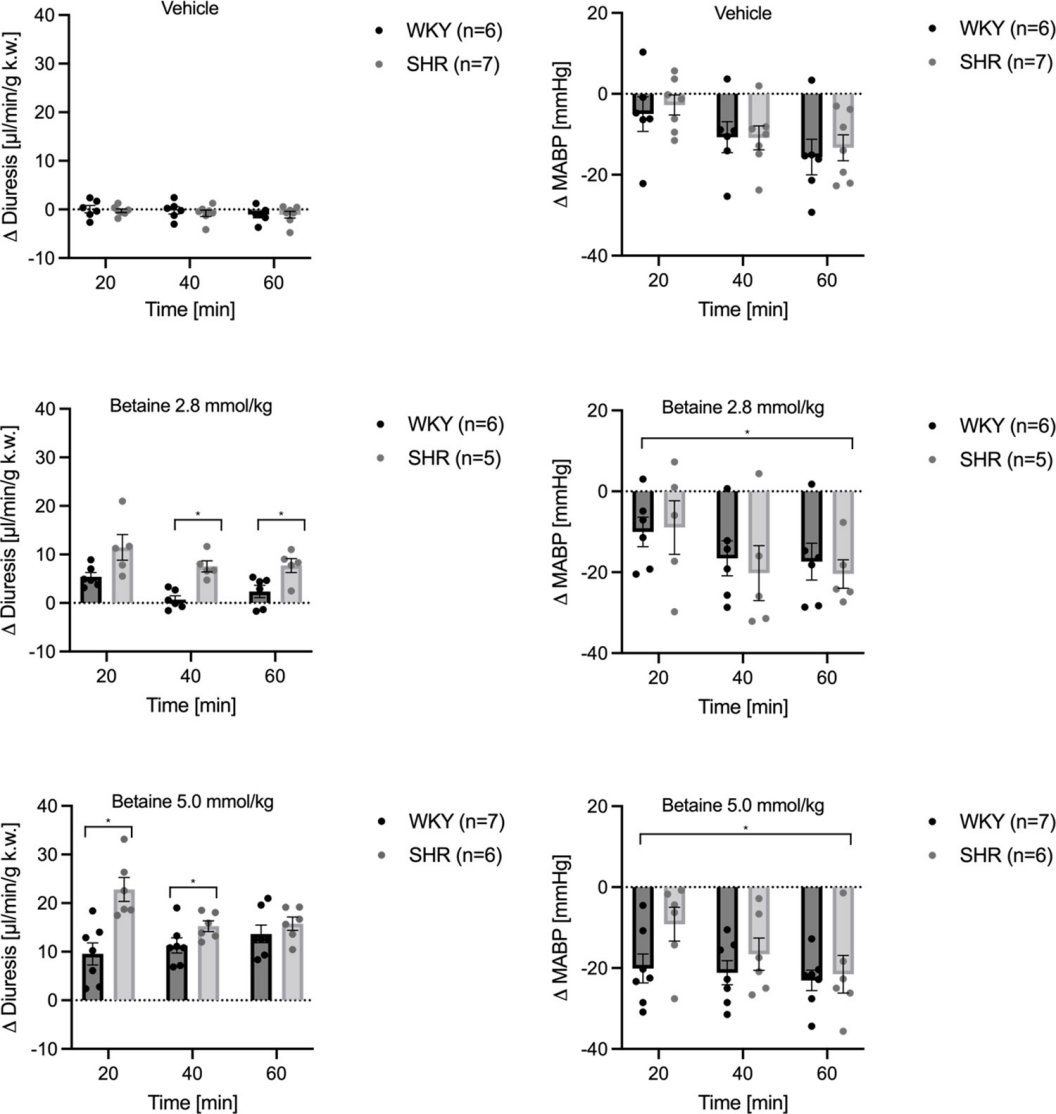
The differences between WKY and SHR responses to betaine. Changes in diuresis [μl/min/g k.w] and mean arterial blood pressure (MABP) [mmHg] in anesthetized Spontaneously Hypertensive rats and Wistar Kyoto normotensive rats following intravenous (IV) infusion of either vehicle or betaine at the dose of 2.8 mmol/kg b.w. or 5.0 mmol/kg b.w. Values are means ± S.E.M, *—p<0.05, ANOVA for repeated measures followed by a t-test for independent variables.

The vehicle or betaine at a dose of 0.5 mmol/kg did not result in changes in diuresis. The administration of betaine at a dose of 2.8 and 5 mmol/kg significantly increased diuresis in both, WKY and SHR (Fig 2). The diuretic response was dose-dependent. Also, the diuretic response to betaine administration was more pronounced in SHR rats than in WKY. Two-way ANOVA (group x time) showed a significant difference between WKY and SHR receiving the same dose of betaine in minutes 20, 40, and 60 (p<0.001) of the experiment (Fig 3).

Betaine significantly increased diuresis and total solute excretion. Results are summarized in Fig 2 and S5 Table.

There was no evident relation between MABP and diuresis changes over time.

## Discussion

Our results demonstrate that hypertensive rats have lower betaine tissue content than normotensive animals. Notably, hypertensive rats excrete greater amounts of betaine with urine. Acute experiments demonstrate that betaine exerts diuretic properties without significantly lowering blood pressure. The diuretic effect of this osmolyte is more pronounced in hypertensive than normotensive rats.

Betaine, an organic osmolyte, protects mammalian cells from high osmotic and hydrostatic stress by helping maintain cell volume [1,2]. Epidemiological studies associate low plasma levels of betaine with elevated BP [20,25,26]. However, data are limited, and the mechanism by which betaine concentrations are reduced in HTN remains unknown. To our knowledge, our study is the first to evaluate levels of betaine in tissues of normotensive and hypertensive animals. The liver is a major site of betaine production [27]. Here, we show that SHRs have significantly lower liver levels of betaine than WKY, which could have contributed to its lower concentration in blood serum in hypertensive rats. Strikingly, despite lower serum concentration of betaine, SHR showed higher 24-hour betaine urine excretion, which may have added to its lower blood levels and tissue depletion of this osmolyte.

We also found that betaine accumulates in all analyzed tissues, with the liver being its largest reservoir in rats. These findings are in line with previous studies [21,22]. Blood concentrations were lower than in other examined tissues except for the brain. Since betaine works as an osmolyte to counterbalance high osmotic and hydrostatic pressure in the tissues [28], we expected significant betaine accumulation in the tissues exposed to such conditions. Indeed, we detected its exceptionally high levels in the renal medulla.

The relationship between urine betaine levels and hypertension has not been investigated before. The present study aimed to fill this gap. Our data suggest that urinary excretion of betaine, which in normal conditions is extremely low and stable [13], increases in hypertension. The glomeruli filter betaine, but almost all of it is reabsorbed in the tubules. Only ~ 5% of the filtered betaine is excreted in the urine [13,14]. In this study, SHRs showed increased urine betaine excretion rate compared to WKYs rats. Previously, it was described that hypertensive rats had altered proximal tubule reabsorption [29] and dysregulated electrolyte balance [30]. Further, excessive osmolytes excretion has been described in kidney disease [31]. To test whether increased betaine content in urine of hypertensive animals was associated with an overt impairment of kidney function or purely hypertension, we analyzed 24-hour urine output and serum creatinine as an estimate for renal function. The analysis showed that although SHR rats had hypertension and higher 24-hour urine output, they did not have significantly altered serum creatinine, which is typical for an early stage of hypertension.

Increased urinary flow in hypertension and consequently reduced reabsorption from the tubule could have accounted for the defective betaine handling in this disease. However, we hypothesized that hypertension might also be associated with abnormal expression of transporters that move betaine across the nephron, as previously described in CKD [31] and DM [32]. To test this, we analyzed the expression of two carrier proteins, namely SLC6a12 betaine/GABA transporter-1 (BGT1) [33–35] and SLC6a20 [32,34,36], since they are the most commonly associated with betaine transport in the kidney. SLC6a20, located in the proximal tubule, is responsible for the reabsorption of imino-acids, such as betaine and L-proline, from the tubular lumen [37]. Our study showed no significant difference in the expression of SLC6a20 mRNA and protein between normotensive and hypertensive rats, which suggests that other mechanisms might be responsible for increased betaine excretion in HTN.

Furthermore, SLC6a12 (BGT1) carrier has been previously described on the basolateral membrane of the medullary cells. Here, contrary to other researchers [34,38], we also detected SLC6a12 mRNA and protein in the kidney cortex. Its mRNA expression in the cortex tends to be higher in SHR than in WKY rats. This might suggest a potential involvement of SLC6a12 in the transport of betaine to the tubular lumen and requires further research.

Another novelty of this study are the diuretic properties of betaine. We previously reported that another osmolyte, trimethylamine N-oxide has diuretic properties [24]. Betaine is a precursor of TMAO [39] and both molecules have a similar structure and molecular weight. We hypothesized that betaine also exerts diuretic properties. IV betaine, but not saline given at the same volume, significantly increased diuresis in anesthetized rats in a similar manner to the previously reported diuretic effect of TMAO [24]. The acute response of SHR rats to betaine load was greater than that of WKYs. We hypothesize that this phenomenon might be a result of dysregulated betaine handling in hypertension. Specifically, increased filtrate production, resulting in increased urinary flow, would shorten the time for betaine reabsorption in the tubules, further contributing to increased diuresis in hypertensive rats.

Finally, our study provides evidence for the lack of significant hemodynamic effects of betaine in normotensive and hypertensive rats. Those results are consistent with previous experimental studies, which found no significant hypotensive effect of betaine supplementation in SHR and WKY rats [40]. Epidemiological studies also support these claims [41].

In summary, hypertension leads to dysregulated water-electrolyte balance [30] and organ damage, including hypertensive nephropathy, which contributes to increased mortality. Studies on the role of osmolytes in hypertension are scarce. Our study shows that in HTN, urinary excretion of betaine increases while its tissue concentration is decreased. Mammalian cells accumulate betaine, among other organic osmolytes, to maintain normal cell function [2]. We hypothesize that depletion of betaine in tissues may contribute to hypertension-related complications. This would be especially important to the kidneys, which are exposed to significant osmotic stress. Further longitudinal studies are needed to evaluate the protective effects of betaine in cardiovascular disease.

### Limitations

This study was only conducted on male rats; thus, it cannot be entirely ruled out that different results could be obtained with female animals. Also, We have observed within-strain differences in baseline total solute excretion (S5 Table), which may be attributed to the inherent biological variability between individuals within the strains or other undetermined environmental factors. Although we cannot provide a definitive explanation for this particular variance, it could be an area for further investigation to better understand the underlying causes of such discrepancies. Further, reabsorption of betaine might be carried out by transporters other than SLC6a20 and SLC6a12, which were investigated in this work. However, the exact set of transporters responsible for betaine reabsorption is not exactly determined yet. Finally, we did not measure betaine concentrations in the blood or kidneys following acute betaine infusion in this study. Also, to prevent any potential hemodynamic alterations, we chose not to perform baseline blood sampling. Therefore, we cannot evaluate the extent to which plasma betaine levels increased due to the infusion. Exploring this avenue in future studies would help determine whether betaine depletion contributes to hypertension or renal damage.

## Conclusion

In conclusion, this may be the first study to report increased betaine excretion in hypertension in the absence of chronic kidney disease and depleted betaine levels in hypertensive animals. In addition, we provide evidence that betaine, similarly to other osmolytes such as urea or TMAO, has diuretic properties.

## Supporting information

S1 Fig Diuresis and betaine excretion in experimental groups at baseline. WKY–Wistar Kyoto rats; SHR–Spontaneously Hypertensive Rats. Values presented are means ± S.E.M. *- p<0.05 vs WKY, by t-test.
(TIFF)

S1 Table Parameters used in HPLC-MS. Monitored transitions for analytes, cone voltages, collision energies, retention times and LOQ of analyzed compounds.
(DOCX)

S2 Table List of oligonucleotide primers used for RT-qPCR. (DOCX)

S3 Table List of antibodies used for Western blot analyses. (DOCX)

S4 Table Baseline hemodynamic parameters in rats. Mean arterial blood pressure (MABP, mmHg) and heart rate (HR, beats/min) at baseline in normotensive Wistar Kyoto rats (WKY) and Spontaneously Hypertensive rats (SHR). Values presented are means ± S.E.M.
(DOCX)

S5 Table Total solutes excretion UOsmV [μosmol/min/g k.w.]. Values presented are means ± S.E.M. U1 –pre-treatment, U2–30 min post-treatment, U3–60 min- post-treatment. P-values calculated with ANOVA, *—p<0.05 post- vs. pre-treatment by Tukey’s post-hoc test.
(DOCX)

S1 Appendix Raw data for all data presented. Raw data includes betaine tissues concentration, metabolic parameters, molecular biology studies, hemodynamics and diuresis measurement.
(XLSX)
