# Supplementary material for: Hypertensive rats show increased renal excretion and decreased tissue concentrations of glycine betaine, a protective osmolyte with diuretic properties
Source: PLoS One. 2024 Jan 2;19(1):e0294926. doi: 10.1371/journal.pone.0294926 (PMC10760924; doi:10.1371/journal.pone.0294926)
Supplement: S1 Table — Monitored transitions for analytes, cone voltages, collision energies, retention times and LOQ of analyzed compounds. (DOCX) [file pone.0294926.s002.docx]

**S1 Table.** **Parameters used in HPLC-MS.** Monitored transitions for analytes, cone voltages, collision energies, retention times and LOQ of analyzed compounds.

| Analyte | MRM transition | Cone voltage [kV] | Collision energy | Retention time [min] |
| --- | --- | --- | --- | --- |
| Betaine | 118.09> 58.09 (qt)  118.09> 59.09 | 20  20 | 40  40 | 1.33 |
| Betaine-d3 | 121.11> 61.06  121.11> 62 (qt) | 20  20 | 15  15 | 1.33 |
