# Supplementary material for: Hypertensive rats show increased renal excretion and decreased tissue concentrations of glycine betaine, a protective osmolyte with diuretic properties
Source: PLoS One. 2024 Jan 2;19(1):e0294926. doi: 10.1371/journal.pone.0294926 (PMC10760924; doi:10.1371/journal.pone.0294926)
Supplement: S2 Table — (DOCX) [file pone.0294926.s003.docx]

**S2 Table.** **List of oligonucleotide primers used for RT-qPCR.**

| **Gene** | **Encoded product** | **Forward (5’-3’)** | **Reverse (5’-3’)** | **PCR product size [bp]** |
| --- | --- | --- | --- | --- |
| *Gapdh* | glyceraldehyde-3-phosphate dehydrogenase | Unique Biorad Assay ID qRnoCID0057018 | | 115 |
| *Slc6a12* | solute carrier family 6 member 12 | AATGTCATCGGGAGCTTGGG | CAAGAGACCACAGGACACCC | 93 |
| *Slc6a20* | solute carrier family 6 member 20 | TGTGCTACGTGTATGGGCTG | TCCATTCTTAGGCCACTGGG | 348 |
