# Supplementary material for: Hypertensive rats show increased renal excretion and decreased tissue concentrations of glycine betaine, a protective osmolyte with diuretic properties
Source: PLoS One. 2024 Jan 2;19(1):e0294926. doi: 10.1371/journal.pone.0294926 (PMC10760924; doi:10.1371/journal.pone.0294926)
Supplement: S3 Table — (DOCX) [file pone.0294926.s004.docx]

**S3 Table. List of antibodies used for Western blot analyses.**

| **Target**  **protein** | **Primary Ab** | **Dilution** | **Secondary Ab** | **Dilution** |
| --- | --- | --- | --- | --- |
| BGT1/SLC6a12 | Rabbit polyclonal, Abcam  Ab200676 | 1:1 000 | Goat anti-rabbit, Abcam  ab97048-1 | 1:10 000 |
| SLC6a20 | Rabbit polyclonal, Invitrogen  **PA5-104153** | 1:1 000 | Goat anti-rabbit, Abcam  ab97048-1 | 1:10 000 |
| Beta-Actin | Goat polyclonal, Abcam  Ab8229 | 1:2 000 | Donkey anti-goat, Abcam  ab97107 | 1:10 000 |
