# Supplementary material for: Hypertensive rats show increased renal excretion and decreased tissue concentrations of glycine betaine, a protective osmolyte with diuretic properties
Source: PLoS One. 2024 Jan 2;19(1):e0294926. doi: 10.1371/journal.pone.0294926 (PMC10760924; doi:10.1371/journal.pone.0294926)
Supplement: S4 Table — Mean arterial blood pressure (MABP, mmHg) and heart rate (HR, beats/min) at baseline in normotensive Wistar Kyoto rats (WKY) and Spontaneously Hypertensive rats (SHR). Values presented are means ± S.E.M. (DOCX) [file pone.0294926.s005.docx]

**S4 Table. Baseline hemodynamic parameters in rats.** Mean arterial blood pressure (MABP, mmHg) and heart rate (HR, beats/min) at baseline in normotensive Wistar Kyoto rats (WKY) and Spontaneously Hypertensive rats (SHR).

| Group | | MABP | HR |
| --- | --- | --- | --- |
| WKY | | | |
| Vehicle | 76.3 ± 2.9 | 265 ± 11 | |
| Betaine 0.5 mmol/kg b.w. | 77.0 ± 5.6 | 247 ± 14 | |
| Betaine 2.8 mmol/kg b.w. | 87.3 ± 2.3 | 281 ± 16 | |
| Betaine 5.0 mmol/kg b.w. | 84.4 ± 4.9 | 306 ± 26 | |
| SHR | | | |
| Vehicle | 110.7 ± 5.3 | 325 ± 16 | |
| Betaine 2.8 mmol/kg b.w. | 115.2 ± 4.6 | 296 ± 22 | |
| Betaine 5.0 mmol/kg b.w. | 117.2 ± 4.6 | 312 ± 14 | |

Values presented are means ± S.E.M.
