# Supplementary material for: Hypertensive rats show increased renal excretion and decreased tissue concentrations of glycine betaine, a protective osmolyte with diuretic properties
Source: PLoS One. 2024 Jan 2;19(1):e0294926. doi: 10.1371/journal.pone.0294926 (PMC10760924; doi:10.1371/journal.pone.0294926)
Supplement: S5 Table — Values presented are means ± S.E.M. U1 –pre-treatment, U2–30 min post-treatment, U3–60 min- post-treatment. P-values calculated with ANOVA, *—p<0.05 post- vs. pre-treatment by Tukey’s post-hoc test. (DOCX) [file pone.0294926.s006.docx]

**S5 Table. Effect of high-dose betaine and vehicle on renal excretion in anesthetized WKY and SHR rats.**

| Group/time point | U1 | U2 | U3 | p-value |
| --- | --- | --- | --- | --- |
| WKY control | 1.98 ± 0.52 | 1.69 ± 0.32 | 1.13 ± 0.13 | 0.284 |
| WKY betaine 5.0 mmol/kg | 2.50 ± 0.42 | 7.85 ± 1.13* | 8.84 ± 1.46* | **0.006** |
| SHR control | 8.25 ± 1.24 | 7.06 ± 1.33 | 4.83 ± 1.44 | 0.240 |
| SHR betaine 5.0 mmol/kg | 3.12 ± 0.52 | 12.02 ± 1.25* | 10.71 ± 1.24* | **< 0.001** |

Total solutes excretion UOsmV [µosmol/min/g k.w.]. Values presented are means ± S.E.M. U1 – pre-treatment, U2 – 30 min post-treatment, U3 – 60 min- post-treatment. P-values calculated with ANOVA, * - p<0.05 post- vs. pre-treatment by Tukey’s post-hoc test.
